# A pregnant woman with thymoma-associated pure red cell aplasia

**DOI:** 10.1186/s12884-022-05145-5

**Published:** 2022-10-27

**Authors:** Megumi Shibata, Kayoko Kaneko, Nagayoshi Umehara, Hitoshi Matsui, Toshinao Kawai, Hisaya Nakadate, Atsuko Murashimia, Haruhiko Sago

**Affiliations:** 1 Division of Obstetrics, Center for Maternal-Fetal, Neonatal and Reproductive Medicine, National Center for Child Health and Development, 2-10-1 Okura, Setagaya-Ku, Tokyo, Japan; 2 Division of Maternal Medicine, Center for Maternal-Fetal, Neonatal and Reproductive Medicine, National Center for Child Health and Development, 2-10-1 Okura, Setagaya-Ku, Tokyo, Japan; 3 Division of Immunology, National Center for Child Health and Development, 2-10-1 Okura, Setagaya-Ku, Tokyo, Japan; 4 Division of Hematology, National Center for Child Health and Development, 2-10-1 Okura, Setagaya-Ku, Tokyo, Japan

**Keywords:** Severe anemia, Pure red cell aplasia, Thymoma, Pregnancy

## Abstract

**Background:**

Pure red cell aplasia (PRCA) is a hematological disorder characterized by anemia with severe reticulocytopenia caused by a marked reduction in erythroid precursors in the bone marrow. PRCA is known to be associated with pregnancy, but thymoma-associated PRCA during pregnancy is very rare, and its successful management has not been reported.

**Case presentation:**

A 37-year-old primiparous woman with severe anemia was referred to our center at 27 weeks’ gestation. She was diagnosed with PRCA based on bone aspiration findings at 33 weeks’ gestation. Magnetic resonance imaging (MRI) revealed an anterior mediastinal mass 4 cm in size suspected of being thymoma. She was therefore diagnosed with thymoma-associated PRCA during pregnancy. Surgery for thymoma was planned after delivery, since the imaging findings were suggestive of early-stage thymoma (Masaoka stage I or II). With transfusion of a total 3,360 ml of red blood cells (RBCs) during pregnancy, the patient gave birth to a baby girl weighing 2,548 g at 40 weeks’ gestation. The baby showed transient congenital cutaneous candidiasis. The placental pathology revealed subamniotic inflammation with a fungal structure. Treatment with topical anti-fungal cream immediately ameliorated the baby’s skin lesion. Maternal anemia did not improve after delivery; however, the thymoma did not increase in size. At five months after delivery, the mother underwent thymectomy with oral cyclosporine A. A pathological examination revealed Masaoka stage II-a thymoma. She completely had recovered from anemia at six months after surgery. Cyclosporine A treatment was discontinued three years after surgery. Remission has been sustained for four years since surgery.

**Conclusions:**

A very rare case of thymoma-associated PRCA during pregnancy was diagnosed without any subjective symptoms and was expectantly managed, resulting in a good prognosis. Although bone marrow aspiration during pregnancy is an invasive test, it is important to confirm the diagnosis. Conservative management with blood transfusion was possible for early-stage thymoma-associated PRCA during pregnancy. Active surveys, including MRI, for PRCA during pregnancy led to the detection of thymoma at an early stage and the achievement of a preferable pregnancy outcome.

**Supplementary Information:**

The online version contains supplementary material available at 10.1186/s12884-022-05145-5.

## Background

Pure red cell aplasia (PRCA) is a hematological disorder characterized by anemia with severe reticulocytopenia caused by a marked reduction in erythroid precursors in the bone marrow [1]. It is classified into primary or secondary PRCA, the latter of which is associated with autoimmune disorders, lymphoproliferative disorders, thymoma, infection, pregnancy, and other conditions [2]. Although there have been some case reports of secondary PRCA associated with pregnancy [3, 4], reports of secondary PRCA associated with thymoma during pregnancy are rare. The successful management of thymoma-associated PRCA during pregnancy has not previously been reported.

We herein report a pregnant woman with thymoma-associated PRCA who was diagnosed by magnetic resonance imaging (MRI) and conservatively managed with blood transfusion during pregnancy, which resulted in a favorable outcome. We also clarify the notable points concerning the management of PRCA during pregnancy.

## Case presentation

A 37-year-old primiparous woman with severe anemia visited our center at 27 weeks’ gestation. The patient, who had no history of immunological or hematological disorders, had suffered from refractory vaginal candidiasis for one year before pregnancy. Anemia had not been noted at a medical health checkup conducted one year before pregnancy or in routine first-trimester blood tests.

The blood tests at the first visit to our center showed severe macrocytic anemia (hemoglobin: 6.9 g/dl) with a mean corpuscular volume of 114 fl. The serum concentrations of iron, vitamin B12, and folic acid were within normal ranges. The peripheral reticulocyte count was very low compared to her severe anemia (0.6% of peripheral red blood cells). She did not show any clinical or laboratory signs of acute hemorrhaging, hemolysis, or infection. Bone marrow aspiration at 33 weeks’ gestation showed severe erythroblastopenia (Supplement Fig. 1).

**Fig. 1:**
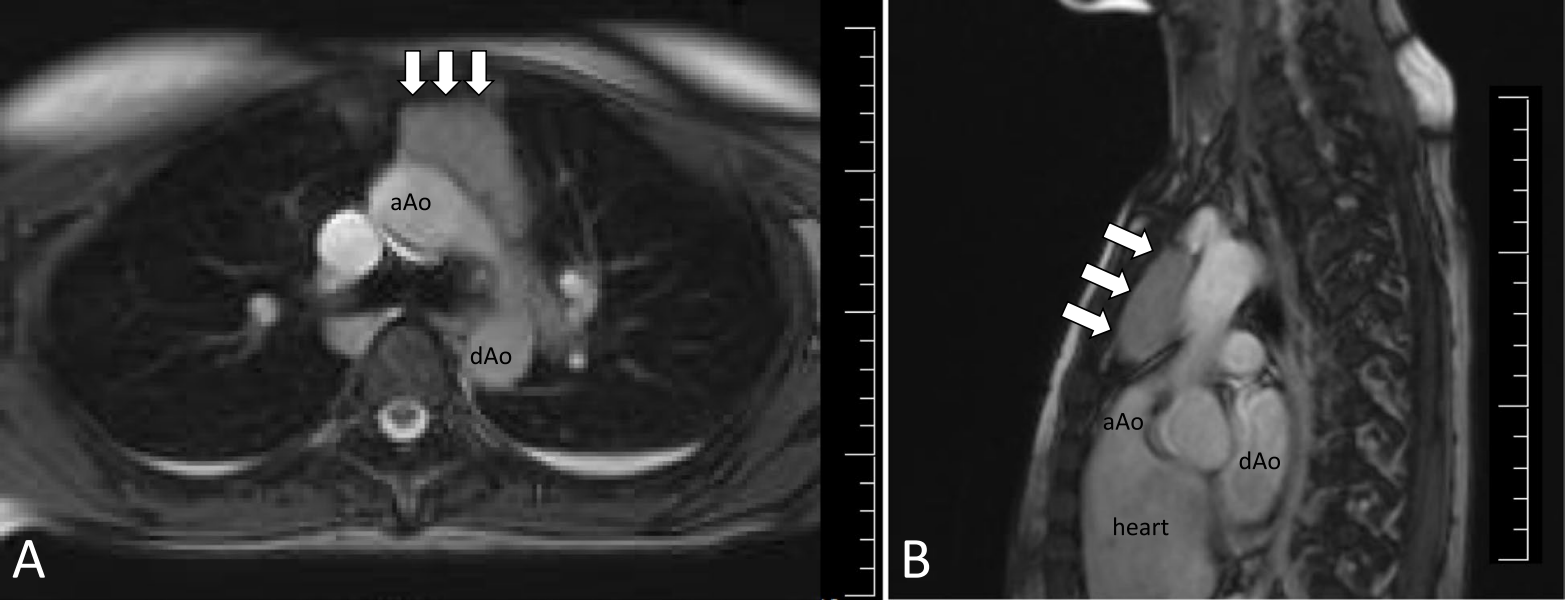
T2-weighted magnetic resonance imaging. **A** Axial view. **B** Sagittal view. The images show a soft tumor mass (marked by white arrows, measuring 46 × 46 × 22 mm) in the anterior mediastinum. aAo, ascending aorta; dAo, descending aorta

MRI revealed a soft tumor mass measuring 46 × 46 × 22 mm in the anterior mediastinum (Fig. 1), even though she had no tumor-related symptoms, such as chest pain, shortness of breath, cough, dyspnea, or suffocation. Finally, we diagnosed the patient with thymoma-associated PRCA, since she had no signs of other causes of PRCA, including lymphoma, leukemia, autoimmune disease, or parvovirus B19 infection.

MRI showed that the margin of the thymoma was smooth, the tumor size was less than 7 cm, and the internal properties were uniform. The mass was not compressing the surrounding internal organs, and there were no signs of local invasion or metastasis to other organs. Accordingly, it was thought to be Masaoka stage I or II (stage I, macroscopically completely encapsulated without capsular invasion; stage II, microscopic invasion into the capsule; stage III, macroscopic invasion into neighboring organs; stage IV, pleural or pericardial dissemination or lymphogenous or hematogenous metastasis) [5]. In the absence of findings suggestive of significant worsening of the condition during pregnancy, we considered postpartum surgery to be acceptable.

The patient required transfusion of a total of 3,360 ml of red blood cells (RBCs; 560 ml each at 32, 33, 34, 38, 40 weeks’ gestation) during pregnancy. She gave birth to a healthy female baby at 40 weeks’ gestation by spontaneous vaginal delivery without any maternal complications. The birth weight was 2,548 g, and the Apgar scores at 1 and 5 min were 8 and 9, respectively. However, diffuse erythematous macular rash was observed over the whole body of the baby, with thrush in her mouth noted just after birth. The skin lesion was suspected to be congenital cutaneous candidiasis, a diagnosis that was confirmed by a thrush culture. The placental pathological findings suggested subclinical candida infection in utero, since *Candida funisitis* was cultured from distinctive pale-yellow plaque on the placenta and umbilical cord (Supplement Fig. 2). The baby’s skin lesion recovered with topical anti-fungal cream treatment.

**Fig. 2:**
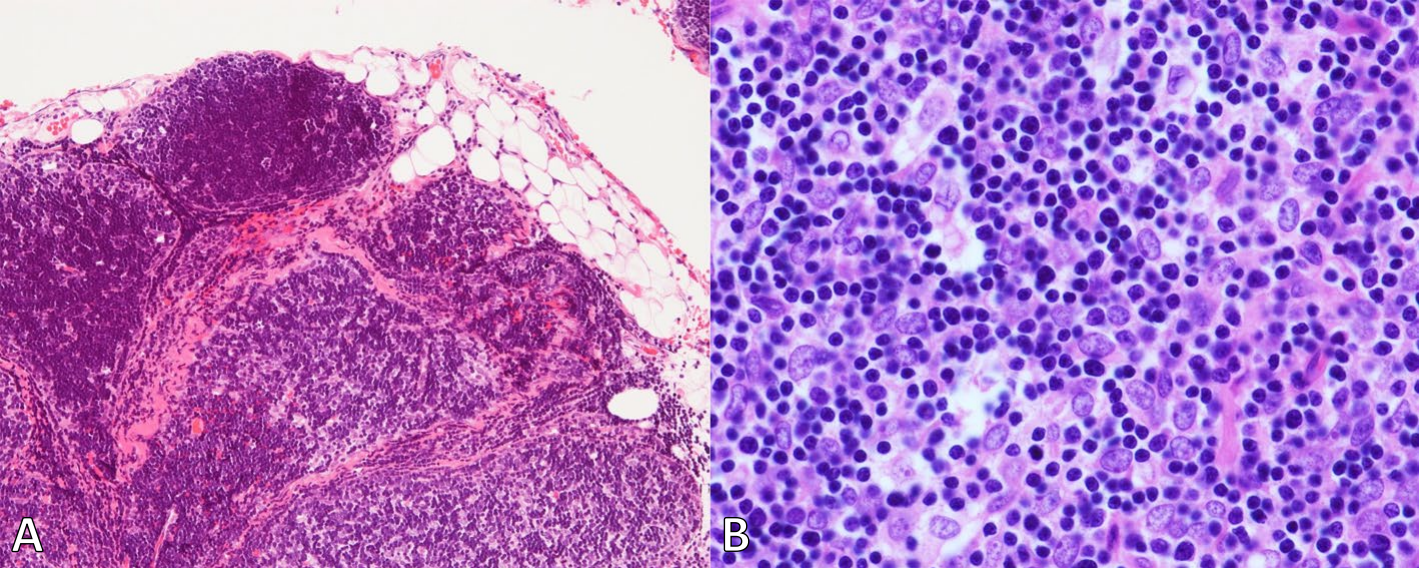
**A** Histopathology of the resected thymoma. Microscopic tumor invasion beyond the capsule was observed, but there was no gross invasion of the thymus or surrounding adipose tissue (marked by black arrows) (Hematoxylin–Eosin staining, magnification × 100). **B** Histopathology of resected thymoma. The thymoma, which showed a cortical thymic epithelial appearance, appeared as interspersed oval cells with pale round nuclei (marked by white arrows) (Hematoxylin–Eosin staining, magnification × 400). The type of equipment was Digital Sight Cameras DS-Ri1, and the Histopathology objective lenses at A and B is × 10 and × 40, respectively

In the postpartum period, chest computed tomography showed that the thymoma had not increased in size, and the patient did not present with any symptoms associated with mass effects of thymoma. The maternal anemia did not improve, and the patient ultimately required another 2,800 ml of RBC after delivery before the operation was performed. Thymectomy was performed at 5 months postpartum, and 300 mg per day of oral cyclosporine A was initiated as induction therapy for PRCA. The histopathological classification revealed that the organotypic thymic epithelial neoplasm predominantly showed a cortical thymic epithelial appearance (WHO type B1). Microscopic tumor invasion was observed beyond the capsule, but no gross invasion of the thymus or surrounding adipose tissue was observed. Accordingly, the patient was diagnosed with Masaoka stage II-a thymoma (Fig. 2).

At 1 month after surgery, her hemoglobin level had increased to 8.9 g/dl without RBC transfusion. Her hemoglobin level improved to 10.4 g/dl at 2 months after surgery and 12.0 g/dl at 6 months after surgery. Three years later, treatment with cyclosporine A was withdrawn. Her hemoglobin level was sustained within the normal range without medication at four years after surgery.

## Discussion and conclusions

We reported a case of thymoma-associated PRCA that was found in the second trimester of pregnancy. Since the thymoma was diagnosed at an early stage according to the Masaoka classification system, we were able to manage it conservatively during pregnancy, which resulted in good outcomes for both the mother and her baby. Although PRCA is known to develop in association with pregnancy, investigating other possible etiologies in PRCA cases found during pregnancy is very important for proper management.

Secondary PRCA diagnosed during pregnancy is classified into two groups based on its etiology: pregnancy-associated PRCA and PRCA induced by other causes. Pregnancy is considered the most common cause of PRCA among cases found during pregnancy. Edahiro et al. reviewed 15 patients with 21 events of pregnancy-associated PRCA in 2020. These cases were treated with RBC transfusion during pregnancy, and the anemia spontaneously ameliorated after delivery. However, in some cases, it took more than six months for patients to recover [3, 4]. Therefore, for the differential diagnosis of pregnancy-associated PRCA, it is important to observe the changes in the hemoglobin level after delivery.

To our knowledge, there have been five reported cases involving pregnant women with secondary PRCA who had etiologies other than pregnancy (Table 1). The causes in those cases were autoimmune disease (*n* = 3 [6–8]), parvovirus B19 infection (*n* = 1 [9]), and thymoma (*n* = 1 [10]). The patient with thymoma underwent multidisciplinary treatment, including surgery; however, she died one month after delivery. It is crucial to identify the etiology of PRCA, especially in PRCA cases that are found during pregnancy.

**Table 1:** Reported cases of secondary pure red cell aplasia during pregnancy

Case	Age	Etiology	Maternal outcome	Infantile outcome	Year	Reference
1	33	RA	Alive	Alive	1989	[6]
2	36	Sjogren	Alive	Alive	2013	[7]
3	25	AOSD	Alive	NA	2014	[8]
4	30	parvovirus B19	Alive	NA	1995	[9]
5	19	thymoma	Death	Alive	1968	[10]
6	37	thymoma	Alive	Alive	2021	present case

*PRCA* Pure red cell aplasia, *RA* Rheumatoid arthritis, *AOSD* Adult-onset Still’s disease, *NA* Not available

There have been 10 previous reports of thymoma during pregnancy in the relevant English literature (Table 2) [11]. All cases were diagnosed based on subjective mass effect symptoms associated with thymoma. Four cases (cases 5, 7, 8, and 9) died from thymoma [10, 12, 13]. Three cases (cases 4, 6, and 10) were not expected to improve with treatment and did not receive therapeutic interventions [11, 14, 15]. These seven cases were Masaoka stage ≥ III. Two cases (cases 2 and 8) chose therapeutic abortion [12, 16]. Our case was diagnosed during the investigation of severe anemia without mass effect symptoms. The patient was assumed to have Masaoka stage I or II thymoma based on the MRI findings, which indicated a good prognosis. Thymoma may generally require surgery through median sternotomy with general anesthesia, a procedure that would be considered highly invasive for a full-term pregnant woman and her fetus [17]. We inferred that in our case, surgery could be performed during the postpartum period, given the predicted Masaoka stage [18].

**Table 2:** Reported cases of thymoma during pregnancy

				Treatment						
Case	Age	Symptoms	Masaoka stage	Chemotherapy	Radiotherapy	Surgery	Maternal outcome	Infantile outcome	Year	Reference
1	25	Acute respiratory	I	-	-	DP	Alive	Alive	2010	[18]
2	31	Dyspnea	II	-	-	PP	Alive	TOP	1991	[16]
3	25	Dyspnea, Chest pain	II	-	-	PP	Alive	Alive	2012	[11]
4	28	Dyspnea	more than II	PP	PP	BP	Alive	Alive	1984	[14]
5	40	Dyspnea	more than III	-	PP	DP	Death	Alive	1986	[13]
6	23	Fever, Cough	more than III	BP	BP	-	NA	Alive	1992	[15]
7	19	Suprasternal mass	IV	-	DP	DP	Death	Alive	1968	[10]
8	22	Shoulder and arm pain	IV	-	PP	DP	Death	TOP	1974	[12]
9	27	Chest pain, Cough	IV	-	DP	DP	Death	Alive	1974	[12]
10	34	Dyspnea	IV	-	-	-	NA	Alive	2012	[11]
11	37	None	II	-	-	PP	Alive	Alive	present case	

*BP* Before pregnancy, *DP* During pregnancy, *PP* Postpartum, *TOP* Termination of pregnancy

The present case was accompanied by chorioamnionitis, funisitis, and neonatal candidiasis at full term. Although candida chorioamnionitis is rare, congenital cutaneous candidiasis at birth leads to a poor prognosis in preterm infants. In full-term infants, congenital cutaneous candidiasis has been reported to be immediately relieved by topical anti-fungal cream [19–21]. Although the underlying pathology is unknown, a negative effect on the maternal immune system induced by thymoma and/or further immunological alterations associated with pregnancy may be associated with the occurrence of neonatal cutaneous candidiasis.

Although thymoma-associated PRCA during pregnancy is very rare, an early diagnosis and appropriate management are crucial for both the mother and her fetus. In situations where pregnancy-associated PRCA is suspected, it is necessary to make a definitive diagnosis by bone marrow aspiration, even during pregnancy, as a definitive diagnosis of PRCA leads to more appropriate management. Active surveys to differentiate other etiologies from pregnancy are essential. MRI for PRCA during pregnancy can lead to the early detection of thymoma when the Masaoka stage is low; in such cases, surgery can be postponed until after delivery. Although we suggest this management, it is based on a case report without any pathophysiological or statistical assessments. The further accumulation of cases with statistical analyses may aid in assessing the management of PRCA during pregnancy.

## Supplementary Information

**Additional file 1: Supplement Fig. 1.** Bone marrow biopsy shows erythroblastopenia without heteromorphic cells (Giemsa staining, magnification ×200). **Supplement Fig. 2.** The pathological examination of the placenta revealed sub-amniotic inflammation with a fungal structure and candida chorioamnionitis (Grocott staining, magnification ×200).

## Data Availability

The data that support the findings of this study are available from the corresponding author, Kayoko Kaneko, upon reasonable request.

## Abbreviations

PRCA: Pure red cell aplasia
MRI: Magnetic resonance imaging
RBC: Red blood cell
