# Supplementary figures and images for: A pregnant woman with thymoma-associated pure red cell aplasia
Source: BMC Pregnancy Childbirth. 2022 Oct 27;22:795. doi: 10.1186/s12884-022-05145-5 (PMC9608914; doi:10.1186/s12884-022-05145-5)

## Slide 1
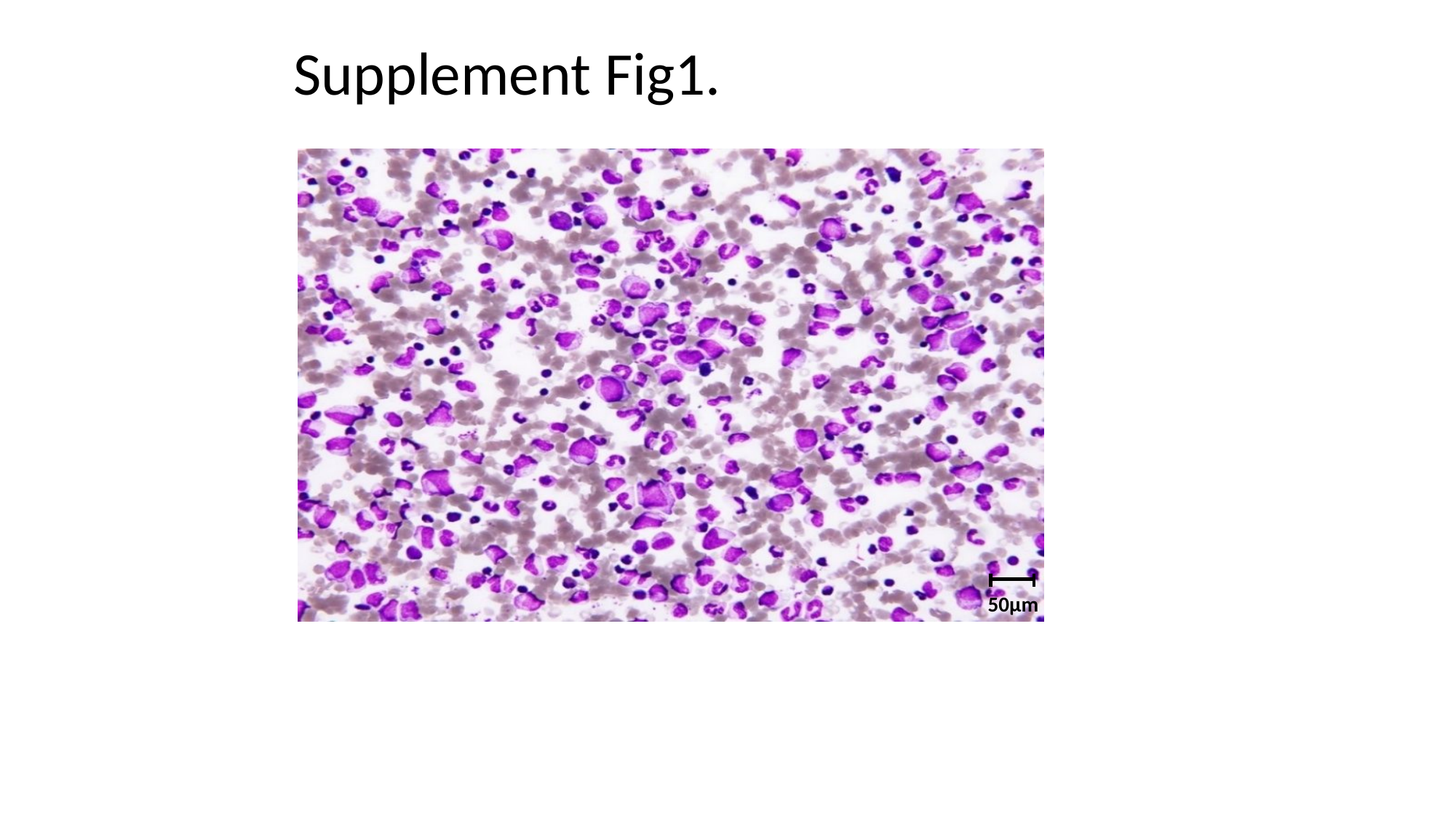

Supplement Fig1.
50μm

## Slide 2
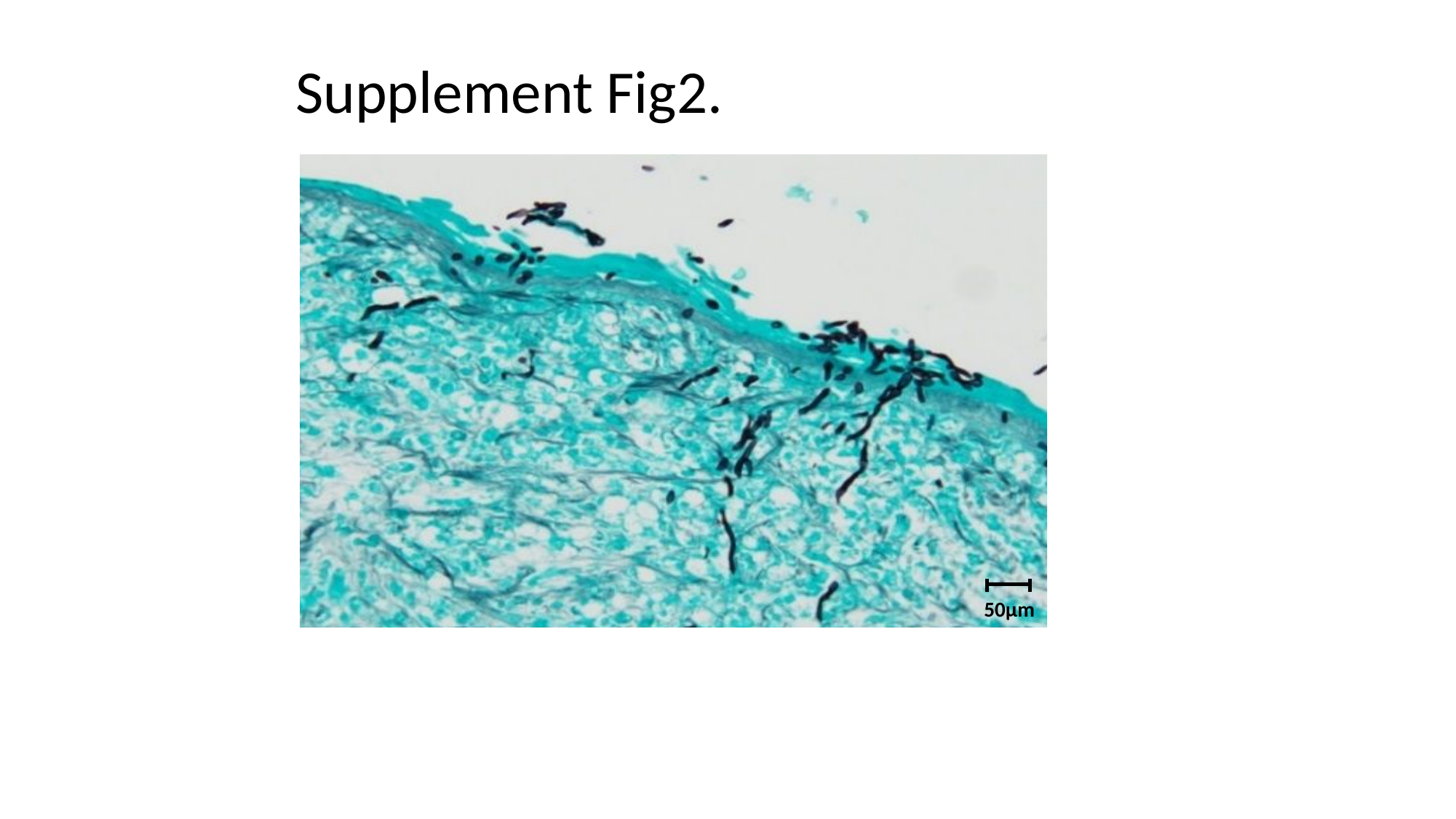

Supplement Fig2.
50μm

Supplement: Supplementary file 1 — Additional file 1: Supplement Fig. 1. Bone marrow biopsy shows erythroblastopenia without heteromorphic cells (Giemsa staining, magnification ×200). Supplement Fig. 2. The pathological examination of the placenta revealed sub-amniotic inflammation with a fungal structure and candida chorioamnionitis (Grocott staining, magnification ×200). [file 12884_2022_5145_MOESM1_ESM.pptx]
